# Engineered Gut Symbiotic Bacterium-Mediated RNAi for Effective Control of *Anopheles* Mosquito Larvae

**DOI:** 10.1128/spectrum.01666-23

**Published:** 2023-07-17

**Authors:** Jinjin Ding, Chunlai Cui, Guandong Wang, Ge Wei, Liang Bai, Yifei Li, Peilu Sun, Ling Dong, Zicheng Liu, Jiaqi Yun, Fang Li, Kai Li, Lin He, Sibao Wang

**Affiliations:** a School of Life Science, East China Normal University, Shanghai, China; b CAS Key Laboratory of Insect Developmental and Evolutionary Biology, CAS Center for Excellence in Molecular Plant Sciences, Institute of Plant Physiology and Ecology, Chinese Academy of Sciences, Shanghai, China; c University of Chinese Academy of Sciences, Beijing, China; d CAS Center for Excellence in Biotic Interactions, University of Chinese Academy of Sciences, Beijing, China; Hubei University of Medicine

**Keywords:** mosquito, dsRNA, RNase III, gut symbiotic bacterium, symbiont-mediated RNAi

## Abstract

*Anopheles* mosquitoes are the primary vectors for the transmission of malaria parasites, which poses a devastating burden on global public health and welfare. The recent invasion of Anopheles stephensi in Africa has made malaria eradication more challenging due to its outdoor biting behavior and widespread resistance to insecticides. To address this issue, we developed a new approach for mosquito larvae control using gut microbiota-mediated RNA interference (RNAi). We engineered a mosquito symbiotic gut bacterium, Serratia fonticola, by deleting its *RNase III* gene to produce double-stranded RNAs (dsRNAs) in the mosquito larval gut. We found that the engineered *S. fonticola* strains can stably colonize mosquito larval guts and produce dsRNAs ds*Met* or ds*EcR* to activate RNAi and effectively suppress the expression of methoprene-tolerant gene *Met* and ecdysone receptor gene *EcR*, which encode receptors for juvenile hormone and ecdysone pathways in mosquitoes, respectively. Importantly, the engineered *S. fonticola* strains markedly inhibit the development of A. stephensi larvae and leads to a high mortality, providing an effective dsRNA delivery system for silencing genes in insects and a novel RNAi-mediated pest control strategy. Collectively, our symbiont-mediated RNAi (smRNAi) approach offers an innovative and sustainable method for controlling mosquito larvae and provides a promising strategy for combating malaria.

**IMPORTANCE** Mosquitoes are vectors for various diseases, imposing a significant threat to public health globally. The recent invasion of A. stephensi in Africa has made malaria eradication more challenging due to its outdoor biting behavior and widespread resistance to insecticides. RNA interference (RNAi) is a promising approach that uses dsRNA to silence specific genes in pests. This study presents the use of a gut symbiotic bacterium, Serratia fonticola, as an efficient delivery system of dsRNA for RNAi-mediated pest control. The knockout of *RNase III*, a dsRNA-specific endonuclease gene, in *S. fonticola* using CRISPR-Cas9 led to efficient dsRNA production. Engineered strains of *S. fonticola* can colonize the mosquito larval gut and effectively suppress the expression of two critical genes, *Met* and *EcR*, which inhibit mosquito development and cause high mortality in mosquito larvae. This study highlights the potential of exploring the mosquito microbiota as a source of dsRNA for RNAi-based pest control.

## INTRODUCTION

Mosquitoes are vectors of various medically important pathogens that cause diseases such as malaria, dengue, Zika, Chikungunya, or West Nile fever, which have a devastating impact on public health and welfare globally (1, 2). A. stephensi, an efficient and invasive vector of urban malaria, originally found in South Asia and the Arabian Peninsula but has been detected in several African countries in the past decade (1). This invasive species is a significant threat to malaria control efforts in Africa (3), as it transmits malaria parasites Plasmodium falciparum and P. vivax with high efficiency and has unique habits, such as outdoor feeding and biting, and resistance to most insecticides, posing a challenge to conventional vector control tools such as insecticide-treated bed nets (ITNs) and indoor residual spraying (IRS) (4). In response to this situation, the World Health Organization has launched a new initiative aimed at halting the spread of A. stephensi in Africa (5) and highlights the urgent need to develop innovative mosquito control strategies. The RNA interference (RNAi) is a highly conserved gene-interfering mechanism that is triggered by double-stranded RNA (dsRNA) homologous to target mRNAs in many eukaryotes (6). RNAi was first discovered in Caenorhabditis elegans (7) and has since been widely used for genetic research in insects (8). Recently, the use of dsRNA designed to silence target gene expression via RNAi has emerged as a promising approach for the development of innovative RNA-based pest management strategies.

The main delivery methods of dsRNA for pest control include microinjection, immersion, oral feeding (9, 10), nanoparticle encapsulation (11, 12), and transgenic plants (13). However, these methods have limited feasibility for large-scale field applications due to the high cost of dsRNA production and its rapid degradation in the environment. Recent studies have explored alternative approaches for delivering dsRNA to mosquitoes, such as using transgenic nonsymbiotic bacteria, yeast, or algae (14–16). However, these studies used microorganisms that cannot colonize the mosquito gut, resulting in transient gene knockdown and limited sustainable effects of RNAi technology. Therefore, there is a critical need for developing a high-efficiency, cost-effective, and sustainable dsRNA delivery system to fully exploit the potential of RNAi technology for mosquito larval control.

In this study, we report a novel approach for dsRNA delivery using gut symbiotic bacterium, which possess the unique characteristic of being able to maintain stable symbiosis within the host and transmits horizontally and vertically, thus achieving sustained action (17–19). We engineered the natural gut symbiotic bacterium Serratia fonticola to produce dsRNAs under the control a constitutive T5 promoter and silence host genes expression in the mosquito larval gut. We demonstrated that the expression of the target genes *EcR* or *Met* in the mosquito larvae were efficiently suppressed by up to 83%, leading to retarded larval development and increased mortality by up to 70%. Our study opens up avenues for the development of symbiont-mediated RNAi (smRNAi)-based approach in more effective and sustainable biocontrol of mosquitoes and other insect pests.

## RESULTS

### Silencing efficiency of the ds*Met* and ds*EcR* in A. stephensi.

**FIG 1:**
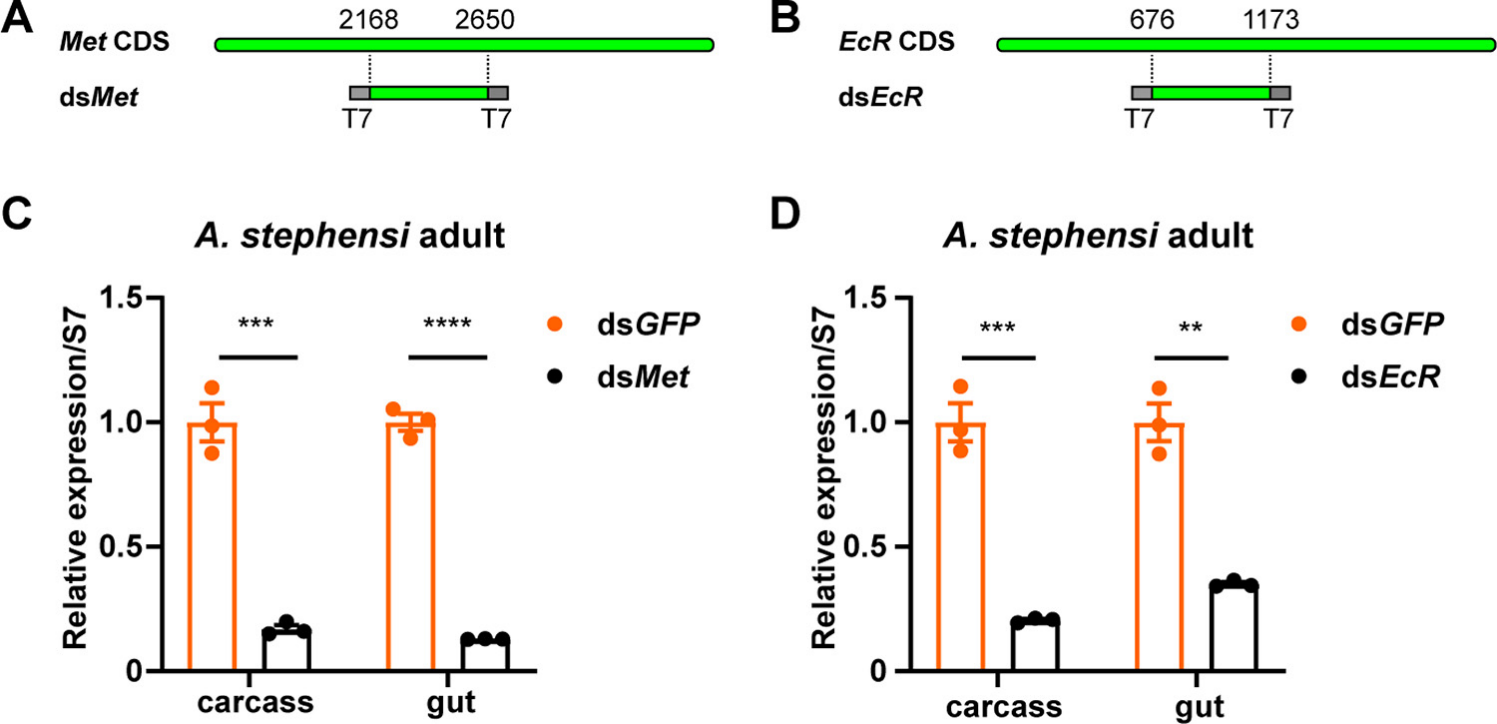
Silencing efficiency of dsRNA targeting *Met* and *EcR* in A. stephensi. (A and B) Schematic representation of dsRNA fragments corresponding to the coding sequence (CDS) region of *Met* or *EcR*. T7 promoter was used to transcribe dsRNA. (C and D) Female A. stephensi adults were injected with ds*Met* or ds*EcR*, while dsRNA targeting GFP (ds*GFP*) served as a negative control. The RNAi efficiency was assessed in the carcass and gut of injected mosquitoes by qPCR at 3 days postinjection. The ribosome gene S7 was used as the reference gene. The expression values were normalized to ds*GFP*. The statistical significance was calculated using Student's *t* test. **, *P < *0.01; ***, *P < *0.001; ****, *P < *0.0001.

Mosquito growth and development are regulated by the coordinated action of juvenile hormones (JH) and ecdysone (20E) (20, 21). Dysregulation of JH and 20E can cause in development retardation, molting or pupation failure, and even mortality (22). *Met* and *EcR* are nuclear receptor genes encoding receptors for JH and 20E, respectively, and have been selected as RNAi targets. By conducting blast analysis of the A. stephensi protein database we were able to identify *Met* and *EcR*. We designed dsRNA corresponding to exon regions of *Met* or *EcR* using the E-RNAi web service (23). Fragments of approximately 500 bp of *Met* or *EcR* were amplified by PCR from the cDNA and used as the templates for generating dsRNA with the T7 transcription systems (Fig. 1A and B). To evaluate the silencing efficiency of the dsRNAs on the expression level of their target genes *Met* and *EcR*, we injected the synthesized dsRNAs into the hemocoel of A. stephensi mosquitoes. We collected carcass and gut tissues from treated mosquitoes to evaluate the silencing efficiency of dsRNA. We found that *Met* transcript levels were reduced by 83% and 87% in the carcass and gut of ds*Met*-treated mosquitoes, respectively, compared to ds*GFP*-treated mosquitoes (Fig. 1C). Meanwhile, *EcR* expression levels were decreased by 79% and 65% in the carcass and gut (Fig. 1D) of ds*EcR*-treated mosquitoes, respectively. These results indicate that the dsRNAs designed for *Met* or *EcR* genes have high silencing efficiency in A. stephensi mosquitoes.

### Larvicidal activity of ds*Met* and ds*EcR*.

**FIG 2:**
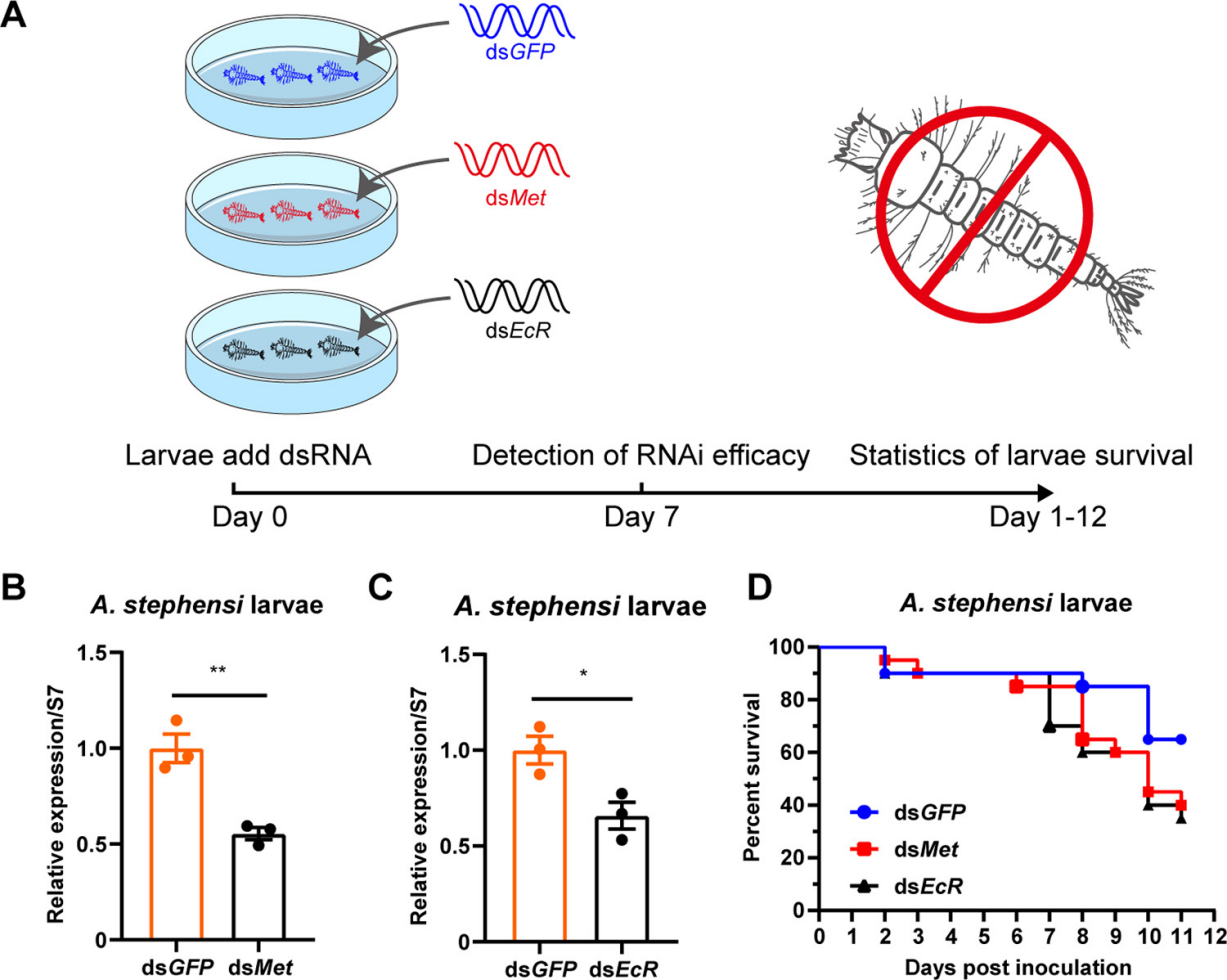
Effects of ds*Met* or ds*EcR* dsRNAs on A. stephensi larval survival and gene expression. (A) Schematic diagram of the study design. Fifty first-instar larvae were reared in 90 mm petri dishes and treated by adding a final concentration of 10 μg/mL of dsRNA targeting *EcR* or *Met* to water. dsRNA of *GFP* (ds*GFP*) was used as a negative control. Larval survival was monitored daily. (B and C) The expression levels of *Met* or *EcR* in larvae were tested by qPCR at 7 days post-dsRNA treatment. *Met* or *EcR* expression levels were normalized to the ribosome gene S7. The expression values were normalized to ds*GFP*. The statistical significance was calculated using Student's *t* test. *, *P < *0.05; **, *P < *0.01. (D) Survival curves of A. stephensi larvae following treatment with ds*Met* or ds*EcR*.

We conducted an assessment of the RNAi and bioeffects of ds*Met* and ds*EcR* on mosquito larvae. First-instar A. stephensi larvae were cultured in petri dish with water containing 10 μg/mL dsRNA, and the number of mosquito larvae was counted daily to evaluate the RNAi effects (Fig. 2A). After 7 days of incubation, total RNA was extracted from A. stephensi larvae, and RT-PCR analysis was performed to investigate the RNAi effects of ds*Met* and ds*EcR*. We found that *Met* expression levels in ds*Met*-treated larvae were 55% of those in ds*GFP*-treated larvae, while the *EcR* transcript levels in ds*EcR*-treated larvae were 66% of those in ds*GFP*-treated larvae (Fig. 2B and C). Furthermore, we observed that ds*Met* or ds*EcR*-treated larvae showed weaker capability and higher mortality compared with ds*GFP*-treated larvae (Fig. 2D). These findings suggest that dsRNAs of *Met* or *EcR* exhibit high silencing efficiency and larvicidal effect against A. stephensi in water. Suppression of *Met* and *EcR* genes by dsRNA led to development retardation and increased mortality of A. stephensi larvae.

### Selection of Serratia fonticola as a mosquito gut symbiont for dsRNA delivery system.

**FIG 3:**
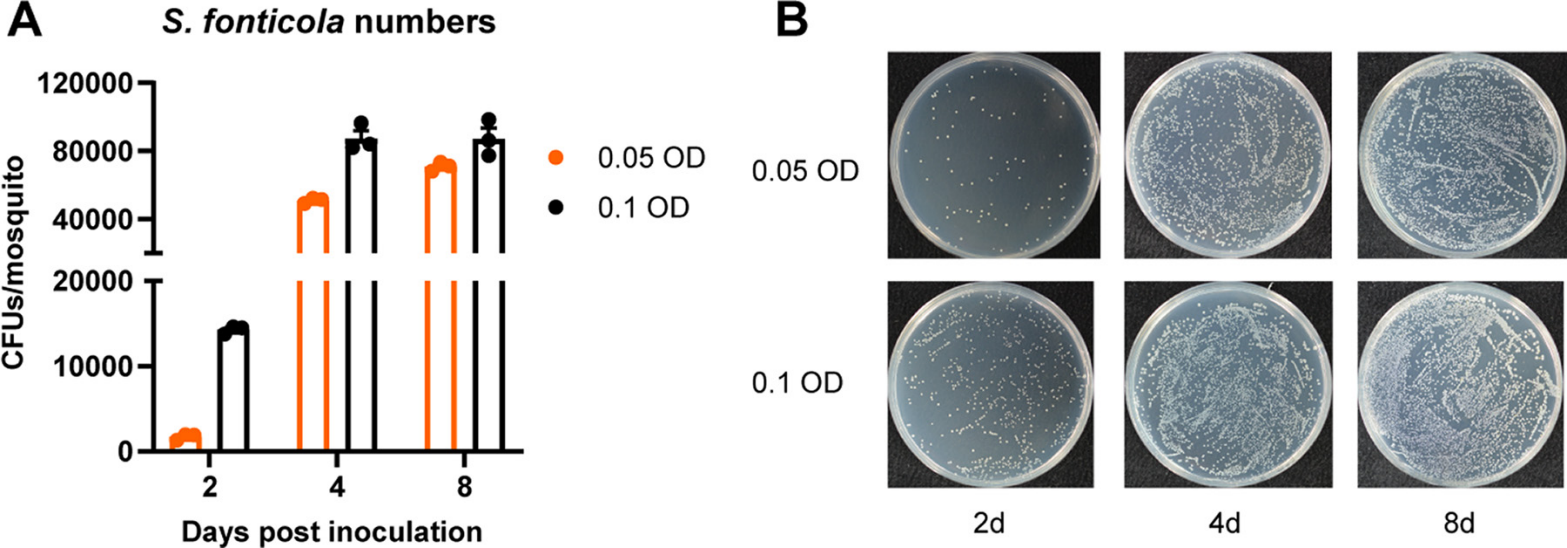
Colonization of *S. fonticola* in A. stephensi larvae. (A) A. stephensi larvae were exposed to GFP-labeled *S. fonticola* at a concentration of 0.05 OD or 0.1 OD in their living water. The guts of mosquito larvae were dissected and homogenized, and the resulting homogenate was plated with serial dilution onto LB agar plates at 2, 4, and 8 days postinoculation. The CFU of *S. fonticola* in the mosquito larvae gut were counted under fluorescence microscope. The data are presented as mean ± SEM (*n* = 3 independent biological replicates). (B) GFP-labeled *S. fonticola* bacterial load in the mosquito larval gut. Bacterial load was determined by plating larval gut homogenates with 1,000 dilution on LB agar plates supplemented with 100 μg/mL of kanamycin. Representative images are shown.

Previous studies have showed that some gut bacteria can stably colonize mosquito guts (17, 18), making gut symbiotic bacteria an ideal delivery system for potent mosquitocidal dsRNAs. Among these symbiotic microbiota, *Serratia* bacteria are widespread in anopheline mosquitoes and are considered the core species (18). We isolated a *S. fonticola* strain from field-caught *A. sinensis* mosquito in China (18). The analysis of 16S ribosomal DNA sequences confirmed the identity of the bacterium as *S. fonticola* (Fig. S1). This bacterium does not impose any fitness cost on adult A. gambiae and A. stephensi mosquitoes, and it can stably colonize the adult mosquito gut when administered to mosquitoes in a sugar meal. It can also be transmitted vertically from female mosquitoes to their progeny and horizontally from male to female mosquitoes (18). To examine the colonization and survival of *S. fonticola* in A. stephensi larvae, we integrated a fluorescent protein gene coding for enhanced green fluorescent protein (eGFP) into the bacterium genome (Sf-GFP). We then exposed the A. stephensi larvae to different concentrations (0.05 or 0.1 OD) of Sf-GFP bacteria in water, and monitored the colonization of *Serratia* in larval gut at different time points (Fig. 3; Fig. S2). We found that Sf-GFP bacteria efficiently colonized the larval gut, even in the presence of an established microbiota. The bacterial population of Sf-GFP increased significantly from day 2 to day 8 (Fig. 3), indicating that *S. fonticola* can stably colonize A. stephensi larvae.

### Generation of RNase III-deficient *S. fonticola* by CRISPR/Cas9.

**FIG 4:**
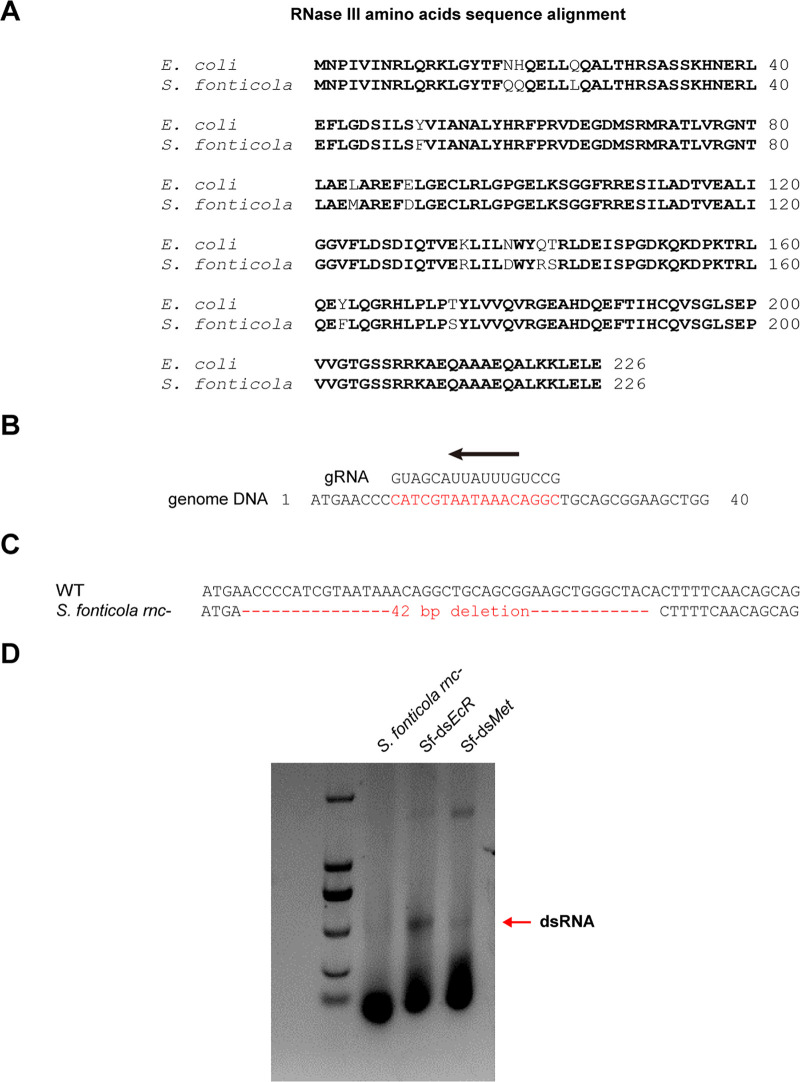
Knockout of *RNase III* in *S. fonticola* using CRISPR-Cas9. (A) Amino acid sequence alignment of RNase III in *S. fonticola* and E. coli. (B) Guide RNA sequences targeting *RNase III* gene in *S. fonticola*. (C) Sanger sequencing indicated a 42-bp deletion in *RNase III* knockout mutant (*S. fonticola rnc*-). (D) Agarose gel electrophoresis showing *Met* or *EcR* dsRNAs produced in *S. fonticola* (Sf-ds*Met* or Sf-ds*EcR*).

To generate dsRNA, *S. fonticola* was genetically manipulated, and an RNase III deficient strain was engineered using the CRISPR/Cas9 system. BLAST analysis of *S. fonticola* genome database allowed the identification of *RNase III* gene (*SfRNaseIII*) with high identity to the Escherichia coli K-12 *RNase III* gene (accession: P0A7Y0) query sequence (Fig. 4A). The predicted *SfRNase III* gene contains 681 nucleotides and encodes a protein of 226 amino acids residues. Analysis of the protein sequence using the CDART tool revealed that SfRNase III belongs to the Rnc Superfamily (24), which is a group of proteins that possess RNase III (RNase III) activity and play a role in RNA processing. We then employed a CRISPR/Cas9-mediated gene editing approach to induce mutations in the open reading frame of *SfRNase III* (Fig. 4B). The Sanger sequencing results showed a 42-bp deletion in *SfRNase III*, which resulted in a N-terminal truncated translation form (Fig. 4C). We designated the resulting strain as *S. fonticola* *rnc-*, which was subsequently used for dsRNA production.

### Production of dsRNA using T5 promoter in *S. fonticola rnc-* strain.

The bidirectional T7 promoter on plasmids have been employed to transcribe dsRNA using T7 RNA polymerase in previous studies (25). However, this method requires the addition of IPTG to induce the translation of T7 polymerase, which is encoded by the plasmids but not present in the E. coli genome (25). In natural settings where IPTG cannot be added, this becomes challenging while using dsRNA-generating symbiotic bacteria in insects. To overcome this limitation, we substituted the T7 promoter with T5 promoter in the dsRNA-expressing plasmid L4440, and the resulting plasmid was designated L4T5 (Fig. S3). T5 promoter can be recognized and used by bacterial RNA polymerase (26, 27).

We separately cloned the dsRNA templates of genes *EcR* and *Met* into L4T5 plasmids and then transformed them into the *S. fonticola rnc-* strain. Subsequently, we investigated the dsRNA production by examining the resulting *S. fonticola rnc-* strains expressing ds*EcR* or ds*Met*, which were referred to as Sf-ds*EcR* or Sf-ds*Met*. Total RNA from Sf-ds*EcR* and Sf-ds*Met* was subjected to agarose/TAE gel electrophoresis, and we observed distinct RNA bands corresponding to ds*EcR* and ds*Met*, respectively (Fig. 4D). These results indicate that the mosquito gut symbiotic bacterium *S. fonticola* can be used to produce dsRNA using L4T5 plasmids.

### *S. fonticola* expressing ds*Met* or ds*EcR* effectively kills A. stephensi larvae.

**FIG 5:**
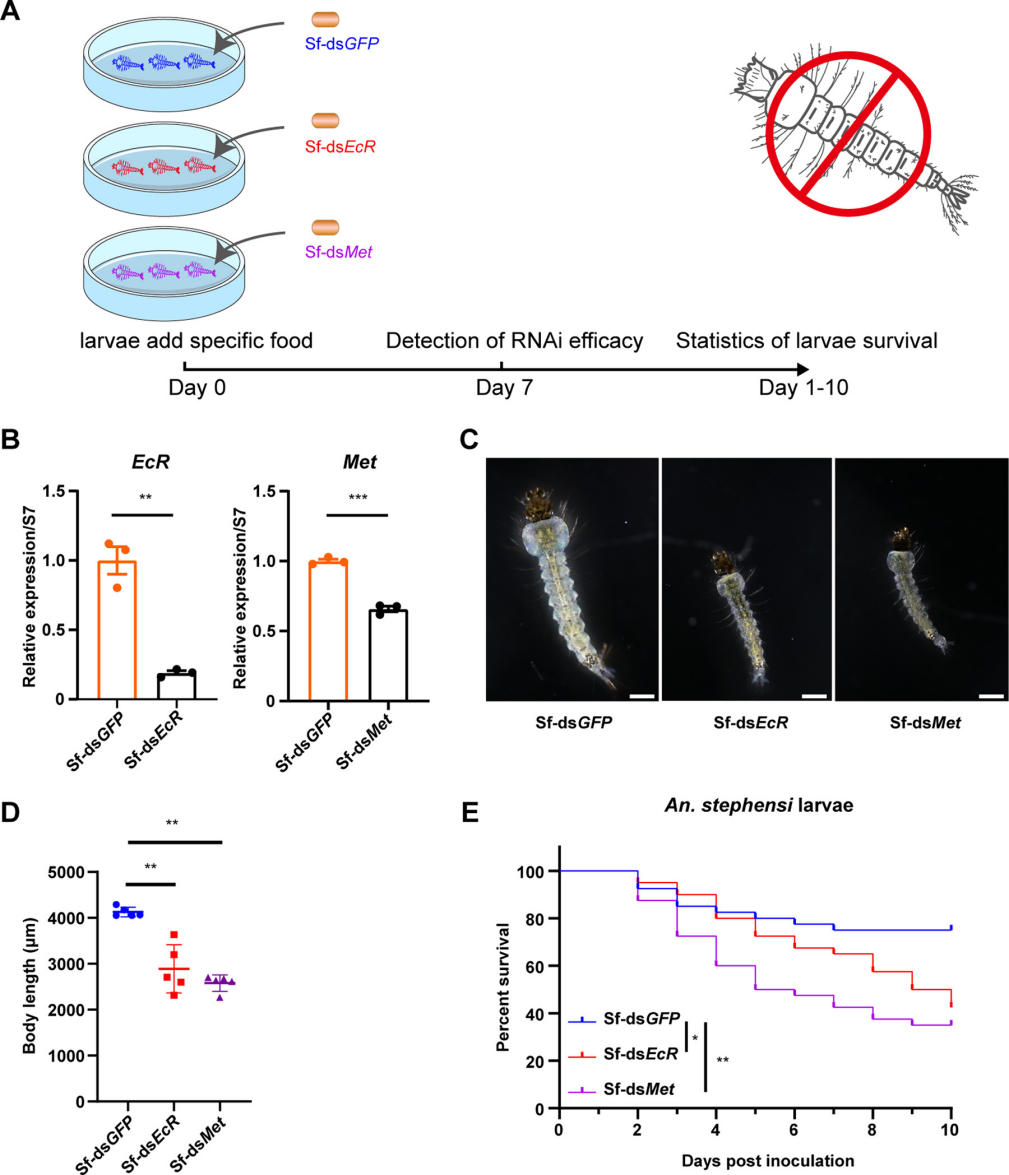
Impact of *S. fonticola* expressing *Met* or *EcR* dsRNA on the growth and survival of A. stephensi larvae. (A) Schematic diagram of the study design. 50 first-instar larvae were reared in 90 mm petri dishes with larval food-coated bacterial granule containing 5 × 10^5^ PFU of *S. fonticola* expressing ds*EcR* (Sf-ds*EcR*), *S. fonticola* expressing ds*Met* (Sf-ds*Met*), or *S. fonticola* expressing ds*GFP* (Sf-ds*GFP*, negative control). Larval survival was monitored daily. (B) qPCR analysis of *Met* or *EcR* expression in larvae at 7 days post addition of larval food-coated bacterial granules. The ribosome gene S7 was served as a reference gene (Student's *t* test). (C) Growth status of A. stephensi larvae fed on larval food-coated bacterial granules containing Sf-ds*EcR*, Sf-ds*Met*, or Sf-ds*GFP*. Scale bar, 500 μm. (D) Body length of A. stephensi larvae fed on larval food-coated bacterial granules (Student's *t* test). (E) Survival curves of mosquito larvae fed on larval food-coated bacterial granules containing Sf-ds*EcR*, Sf-ds*Met*, or Sf-ds*GFP*. The survival data of mosquito larvae were analyzed using a log-rank (Mantel-Cox) test. *, *P < *0.05; **, *P < *0.01; ***, *P < *0.001.

To examine the RNA silencing and biological impact of recombinant *S. fonticola* strains expressing ds*EcR* and ds*Met* on A. stephensi larvae, we developed user-friendly larval food-coated bacterial granules comprising concentrated live Sf-ds*EcR or* Sf-ds*Met* bacteria. This formulation is easy to use, does not disperse in water, and sinks to the bottom of treated containers, where A. stephensi larvae readily consume it (Fig. 5A).

We added 50 mosquito larvae to petri dishes containing food granules. We found that a significant reduction in *EcR* or *Met* expression levels in Sf-ds*EcR* or Sf-ds*Met* treated mosquito larvae compared to the Sf-ds*GFP*-treated group (Fig. 5B). This indicates that the dsRNA generated by *S. fonticola* can trigger RNA interference in mosquito larvae. Importantly, larvae treated with Sf-ds*EcR* or Sf-ds*Met* developed slowly (Fig. 5C), while those treated with Sf-*GFP* developed normally (Fig. 5C). At 10 days posthatching, the body size of Sf-ds*EcR* or Sf-ds*Met* treated larvae was reduced by more than 25% compared to Sf-ds*GFP* treated larvae (Fig. 5D). Moreover, Sf-ds*EcR* or Sf-ds*Met* treatment induced up to 57.5% or 65% larval mortality, respectively, at 10 days posthatching (Fig. 5E). These results demonstrate that *S. fonticola* expressing ds*Met* or ds*EcR* dsRNA inhibits the development of mosquito larvae and effectively kills A. stephensi larvae.

## DISCUSSION

The use of dsRNA for RNAi-mediated degradation of targeted mRNA has shown promise as a novel tool for RNA-based mosquito management strategies (16, 28). However, there are still several challenges that needs to be addressed, such as the high cost of dsRNA production, inconsistent RNAi efficiency, and the nonsustainable delivery methods used to administer dsRNA to mosquitoes. To overcome these challenges, we report an innovative approach that involves using engineered symbiotic bacteria to deliver dsRNA to mosquito larvae. This approach offers a more targeted, cost-effective, efficient delivery, and long-lasting effect of RNAi, while also minimizing environmental impact, and holds promise for the development of novel, long-term mosquito management strategies.

Symbiotic bacteria are known to provide several benefits to their insect hosts, including nutritional supplements, manipulation of host immune homeostasis, and tolerance to environmental perturbations (29). Recent studies have shown that the mosquito gut harbors its microbiome in a selective way (30). *Serratia* bacteria are dominant gut symbionts of anopheline mosquitoes and are commonly present in water, soil, and plant surfaces (18, 31). *S. fonticola*, a ubiquitous inhabitant of aquatic environments, soil, and plants (32, 33), is a possible source of *S. fonticola* for field mosquito larvae. Therefore, *S. fonticola* is an excellent candidate for delivering dsRNA to mosquito larvae in the field.

We showed that a naturally occurring bacterium, *S. fonticola*, from filed-caught mosquitoes can efficiently colonize A. stephensi larval gut and can be engineered to produce dsRNA. We generated an RNase III deficient strain of *S. fonticola* using the CRISPR/Cas9 technique to knock out the *rnc* gene. Instead of using dsRNA expressing plasmids containing bidirectional T7 promoters that require additional IPTG to initiate transcription, we substituted the T7 promoters with T5 promoters that can be recognized and used for transcription by bacteria RNA polymerase. The engineered bacteria transformed with T5-dsRNA plasmids could efficiently generate and accumulate dsRNA in high levels. Furthermore, we developed a larval food-coated granule with concentrated live engineered bacteria and an easy-to-use formulation, which is crucial for future efforts to introduce the engineered bacteria into field mosquito larvae population. Importantly, *S. fonticola*, as a mosquito gut symbiotic bacterium, has the potential to transmit horizontally and vertically within mosquito populations, making a one-time implementation have a long-term effect. These facts make *S. fonticola*-mediated RNAi technology an excellent potential for field mosquito larvae control.

Numerous studies have shown the effectiveness of RNAi in inducing insect mortality by targeting essential genes involved in development, such as *chitin synthase* (34, 35), *tubulin* (36, 37), *Vacuolar-type H^+^-ATPase* (38, 39), etc. In this study, we developed dsRNAs that target the *Met* or *EcR* genes, which encode the JH or ecdysone hormone receptors in insects (40, 41). Dysregulation of JH or ecdysone hormone signaling can lead to abnormal growth and development, reduced reproductive capacity, and even death. Our results demonstrate that the engineered *Sf*-ds*EcR* or *Sf*-ds*Met* can effectively inhibit the development of A. stephensi larvae and induce high larval mortality. In future study, we may consider expressing different sets of dsRNAs that target different genes or pathways important for mosquito development, metabolism, immunity or other physiological processes to further increase RNAi efficacy, providing a powerful tool for mosquito control. However, more work lays ahead before this approach can be implemented in the field. One key issue is addressing regulatory, ethical, and social issues related to the release of genetically modified organisms.

## MATERIALS AND METHODS

### Mosquito rearing.

Anopheles stephensi (Dutch strain) mosquitoes were maintained at 27°C and 75% ± 5% relative humidity under a 12 h/12 h light-dark cycle and fed on 10% (wt/vol) sucrose. The larvae were reared on cat food pellets and ground fish food supplement (17).

### *In vitro* synthesis of double-stranded RNA.

To synthesize double-stranded RNA of the genes *Met* and *EcR*, a 483 bp or 498 bp DNA fragment from the coding region of *Met* or *EcR* was PCR amplified from A. stephensi cDNA with primers supplemented with the T7 promoter sequence at their 5′ ends (5′-TAATACGACTCACTATAGGG-3′) (Table S1). The PCR products were purified with the Cycle-Pure Kit (OMEGA) and used as templates to synthesize dsRNA *in vitro* using the MEGAscript RNAi kit (Life Technologies). The dsRNA was further purified using the purification column supplied with the kit, eluted with nuclease-free water, and concentrated to 3 μg/μL using a Microcon YM-100 filter (Millipore). An enhanced green fluorescent protein (eGFP)-derived double-stranded RNA (ds*GFP*) was synthesized and used as a negative control.

### dsRNA-mediated gene silencing in mosquitoes.

For RNAi in adult mosquitoes, 20 cold-anesthetized 3-day-old female mosquitoes were injected with 69 nl dsRNA solution of *Met* or *EcR* (3 μg/μL) into the hemocoel using Nanoject III microinjector (Drummond). Mosquitoes injected with ds*GFP* were used as negative controls. RNAi efficacy was examined at 3 days postinjection.

For RNAi in mosquito larvae, groups of 50 new hatched first-instar larvae were reared separately in 90 mm petri dishes, and dsRNA of *Met* or *EcR* was supplied into water at final concentration of 10 μg/mL. ds*GFP* was used as negative control. RNAi efficacy was tested at 7 days post-treatment, and mortality was recorded daily.

### Mosquito RNA isolation and qPCR.

Adult mosquito midguts and carcass were dissected in ice-cold PBS and stored in 1.5 mL Eppendorf tubes. Mosquito larvae were collected and transferred to EP tube. Mosquito samples were homogenized after immersion in 500 μL RNAiso plus (TaKaRa). Total RNA was extracted according to the manufacturer’s instruction. cDNAs were synthesized using the Hifair III 1st Strand cDNA Synthesis SuperMix for qPCR (Yeasen). Quantitative reverse transcription-PCR (qPCR) reactions were performed using *Taq* Pro Universal SYBR qPCR Master Mix (Vazyme). Each sample was performed in triplicate. The ribosomal protein gene S7 was used as endogenous control. Primer sequences were listed in Table S1.

### 16s rRNA gene amplification, sequencing, and phylogenetic analysis.

Mosquito gut symbiotic microbiota were investigated in our previous study (18). Bacterial genomic DNA was isolated using the TIANamp Bacteria DNA Kit (Tiangen). The 16S rDNA was PCR amplified using primers 27F and 1492R (Table S1) and purified with the Cycle-Pure Kit (OMEGA). The 16S rDNA sequences were identified by sanger sequencing and aligned using BLAST search in the NCBI database. The phylogenetic tree was constructed using the neighbor-joining method with the MEGA7 software (42).

### Colonization of *S. fonticola* in the mosquito gut.

To test the colonization of *S. fonticola* in mosquito larvae gut, a GFP-labeled strain of *S. fonticola* (Sf-GFP) was used, as previous described (43). The bacteria were cultured in LB broth medium at 30°C, washed twice in 1×PBS, and resuspended in deionized water. Subsequently, Sf-GFP was introduced into newly hatched 1-star larvae in their aquatic habitat at a concentration of OD600 = 0.05 or 0.1 (equivalent to 0.05 OD or 0.1 OD). At 2, 4, and 8 days after the Sf-GFP treatment, 10 larvae were subjected to surface sterilization by washing in 75% ethanol, followed by rinsing in sterile PBS three times. The larvae’s guts were dissected and homogenized in 1× PBS. WT mosquito larvae were used as a control. To determine the colonization of Sf-GFP, 10-fold serial dilutions of the larval gut homogenates were plated on LB agar plates containing 100 μg/mL of kanamycin. The Sf-GFP colonies were counted using fluorescence microscopy.

### Generation of RNase III-deficient *S. fonticola* strain.

To delete the *RNase III* gene in gut symbiotic bacterium, CRISPR/Cas9 mediated gene knock out of RNase III in *S. fonticola* was performed as previous described (44). Briefly, a Cas9 plasmid and a gRNA expressing plasmid were electroporated into *S. fonticola*. Gene deletion was confirmed by PCR. The mutant strain, *S. fonticola rnc*-, was made competent for further transformation of dsRNA expression plasmids.

### Construction of dsRNA expression plasmids.

To avoid using lactose to induce dsRNA production, we constructed a constitutive dsRNA expression plasmid by replacing T7 promoter in L4440 with bacteriophage T5 promoter. The resulting L4T5 plasmid could produce dsRNA without induction. For production of ds*Met* or ds*EcR*, the DNA sequences corresponding to *Met* or *EcR* were PCR amplified from the A. stephensi cDNA, and then subcloned into BglII and KpnI sites of the L4T5 plasmid.

The dsRNA expression plasmids were transformed into the *S. fonticola* *rnc-* mutant. To quantify the production of dsRNA from *S. fonticola*, the bacterial pellet of overnight culture was washed by PBS twice and resuspended in 70% ethanol vol/vol in PBS, incubated at 4°C for 5 min. The pellet collected by centrifugation at 10,000 g for 10 min under 4°C. dsRNA resolved in supernatant was applied to agarose gel electrophoresis (45).

### Feeding of mosquito larvae with dsRNA expressing bacteria.

Overnight cultures of dsRNA expression *S. fonticola* were collected by centrifugation and washed three times with 1× PBS. The collected *S. fonticola* was mixed with agar and cat food powder to make a semisolid larvae food. The elaborate food was cut into small granules and store at 4°C, with each granule containing 5 × 10^5^ PFU of bacteria.

To test the effects of dsRNA expression *S. fonticola* on mosquito larvae, groups of 50 newly hatched first-instar larvae were separately reared in 90 mm petri dishes. One piece of larval food-coated bacterial granule was fed to mosquito larvae every day. RNAi efficacy was tested at 7 days post-treatment. The pictures of mosquito larvae were captured by an Olympus microscope MVX10, and the body length was measured. Survival of larvae was recorded every day.

### Statistical analyses.

Statistical analysis was conducted using GraphPad Prism version 5.00 for Windows (GraphPad Software). Details of the statistical methods are listed in the figure legends. The survival data of mosquito larvae were analyzed using a log-rank (Mantel-Cox) test. Statistical significance between two treatments was determined using an unpaired Student's *t* test. *P* value of < 0.05 was considered statistically significant.
