# Supplementary material for: Engineered Gut Symbiotic Bacterium-Mediated RNAi for Effective Control of Anopheles Mosquito Larvae
Source: Microbiol Spectr. 2023 Jul 17;11(4):e01666-23. doi: 10.1128/spectrum.01666-23 (PMC10433860; doi:10.1128/spectrum.01666-23)
Supplement: Supplemental file 1 — Fig. S1 to S3. Download spectrum.01666-23-s0001.docx, DOCX file, 0.8 MB [file spectrum.01666-23-s0001.docx]

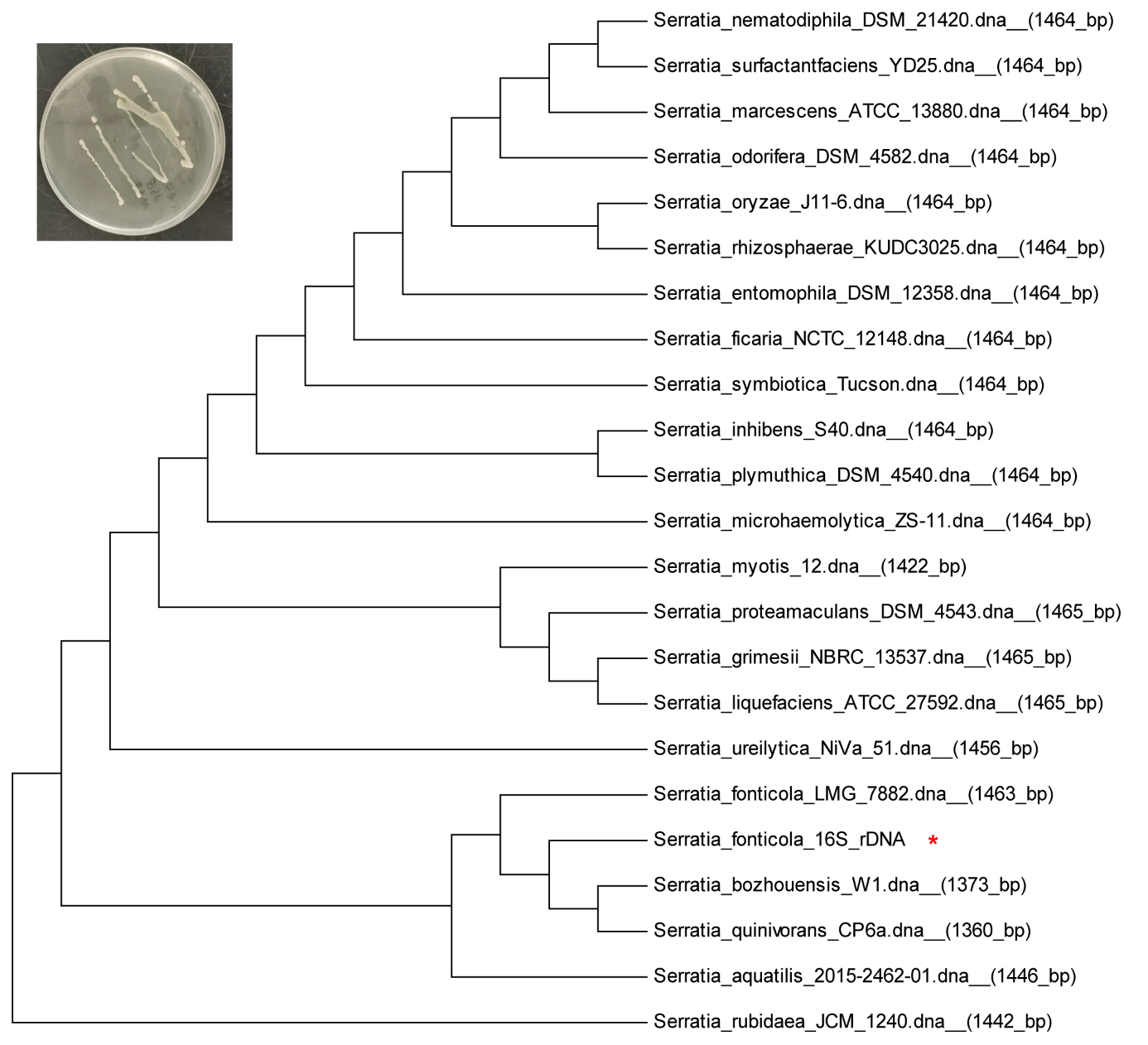


**Figure S1. Phylogenetic analysis of *Serratia fonticola* based on 16S rRNA gene sequences.**

Phylogenetic analysis of *Serratia* bacteria based on 16S rRNA gene sequences was performed using MEGA 7.0.
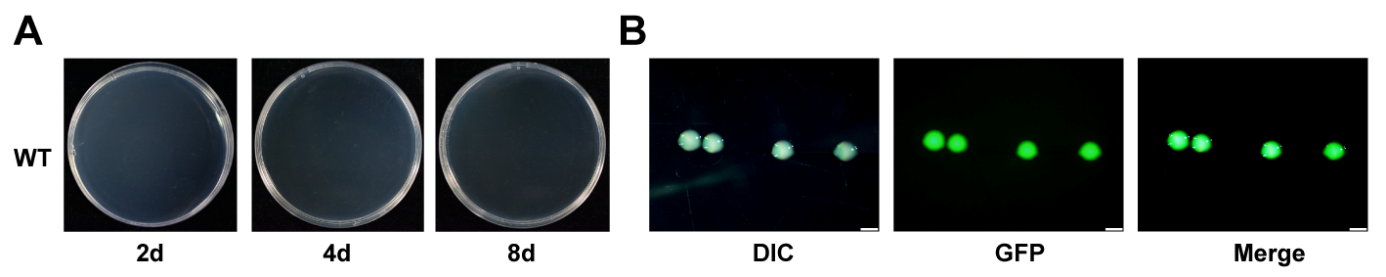


**Figure S2. *A. stephensi* larvae gut microbiota grew on LB agar plates.**

(A) The guts of WT mosquito larvae were dissected and homogenized, and the resulting homogenate was plated with serial dilution onto LB agar plates at corresponding time points. The aborigine’s microbiota in mosquito larvae could not grow on LB agar plates (Kana+). (B) Colonies of GFP-labeled *S. fonticola* (Sf-GFP) grew on LB agar plates. DIC: differential interference contrast microscopy. Scale bars: 1mm.


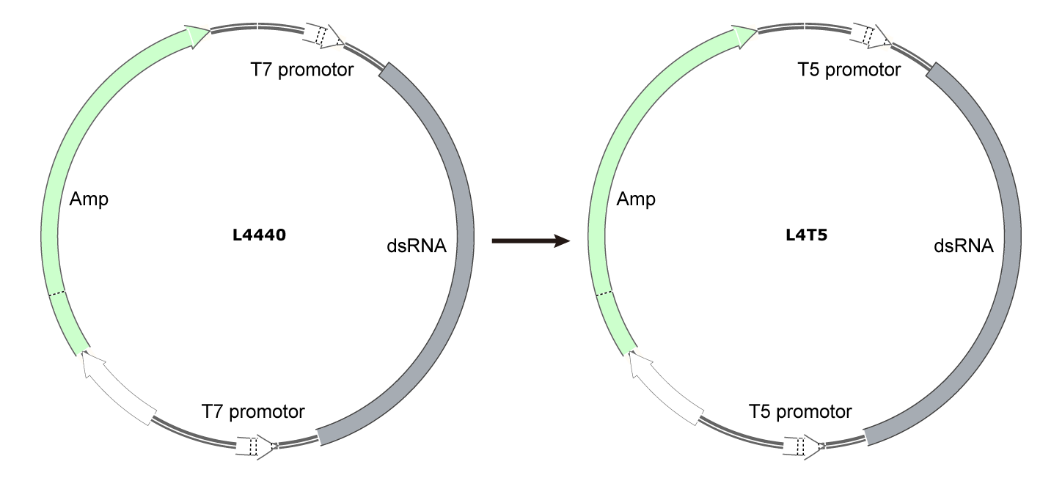


**Figure S3. Construction of *S. fonticola* dsRNA-expressing plasmid.**

Map of the L4T5 plasmid used for dsRNA generation in *S. fonticola*. The T7 promotor in the L4440 plasmid was replaced with the bacteriophage T5 promotor.
